# Apolipoprotein CII: Cornerstone of Pathophysiological Interplay in Aortic Stenosis and type 2 Diabetes Mellitus

**DOI:** 10.14336/AD.2025.0518

**Published:** 2026-06-20

**Authors:** Nerea Corbacho-Alonso, Tamara Sastre-Oliva, Inés Perales-Sánchez, German Hernández-Fernandez, Emilio Blanco-López, Luis F López-Almodovar, Jorge Solis, Luis R Padial, Teresa Tejerina, Ana E Lopez-Jimenez, Laura Mourino-Alvarez, Maria G Barderas

**Affiliations:** ^1^Department of Vascular Physiopathology, Hospital Nacional de Parapléjicos, SESCAM, 45071, Toledo, Spain.; ^2^Department of Vascular Physiopathology, Hospital Nacional de Parapléjicos, IDISCAM, 45071, Toledo, Spain.; ^3^Cardiac Surgery, Hospital General Universitario de Toledo, SESCAM, 45007, Toledo, Spain.; ^4^Department of Cardiology, Hospital Universitario 12 de Octubre and Instituto de Investigación Sanitaria Hospital 12 de Octubre (imas12), 28041, Madrid, Spain.; ^5^AtriaClinic, 28009, Madrid, Spain.; ^6^Centro de Investigación Biomédica en Red de Enfermedades Cardiovasculares (CIBERCV), Instituto de Salud Carlos III, 28029, Madrid, Spain.; ^7^Department of Cardiology, Hospital General Universitario de Toledo, SESCAM, 45007, Toledo, Spain.; ^8^Department of Pharmacology, School of Medicine, Universidad Complutense de Madrid, 28040, Madrid, Spain.; ^9^Department of Clinical Biochemistry, Hospital Universitario 12 de Octubre, 28041 Madrid, Spain.; ^10^Department of Cardiology, Ciudad Real General University Hospital, 13005 Ciudad Real, Spain.

**Keywords:** calcific aortic stenosis, diabetes mellitus, APOC2, personalized medicine

## Abstract

Different studies have demonstrated that the progression from mild to severe calcific aortic stenosis (CAS) is faster in patients with type 2 Diabetes Mellitus (T2DM). Hence, we set out to find markers to improve the clinical management of individuals with both pathologies. Calcified and non-calcified valve tissue from patients with CAS, or with CAS and T2DM, was studied using a multiomics (proteomics and transcriptomics) strategy, highlighting the apolipoprotein CII (apoC2) protein as a potential biomarker. To define its diagnostic potential, we used ELISA and turbidimetry to measure the apoC2 in plasma from an independent cohort of patients. Moreover, an *in vitro* model of aortic valve interstitial cells (VICs) was used to evaluate the therapeutic potential of apoC2. This multiomics study demonstrated the increase in apoC2 protein and APOC2 gene expression in the valves of patients without T2DM. These changes were also reflected in their plasma, highlighting its potential as a diagnostic marker. Finally, exposure to recombinant apoC2 augments calcification and lipid deposition in VICs, effects that were dampened by silencing its expression, emphasizing the potential of apoC2 as therapeutic target. While the existence of T2DM affects the profile of the aortic valve, we have identified apoC2 as a potential diagnostic and therapeutic marker, an important step towards better clinical management that will help slow the progression of CAS in diabetic patients.

## INTRODUCTION

Calcific aortic valve disease (CAS) represents a major public health burden, the prevalence of which is increasing due to the aging of the population. The progressive deterioration and thickening of the aortic valve (AV) associated with CAS prevents the valve from opening properly, and the ensuing narrowing restricts blood flow from the left ventricle to the rest of the body [1, 2]. Usually, CAS remains asymptomatic for long periods of time (up to several years) but once diagnosed, its progression is inevitable, rapid and with a poor prognosis. Currently, there are no pharmacological treatments to delay or prevent CAS, with surgical or transcatheter AV replacement being the only therapeutic options available [3].

CAS and type 2 Diabetes Mellitus (T2DM) are closely related chronic diseases, with numerous studies showing that the prevalence of T2DM is higher in patients with CAS than in the population at large, ranging between 11.4 and 31.6% [4-9]. Moreover, T2DM exacerbates the severity of CAS, exaggerating AV inflammation, oxidative stress and calcification through mechanisms that are not fully understood [10-13]. Consequently, the treatment of CAS becomes more complex when both pathologies concur in the same patient, and when untreated, together, these conditions provoke significant morbidity and mortality. While the evidence available suggests that surgical intervention is appropriate for patients with severe asymptomatic CAS, T2DM is an important risk factor in these patients as it affects both their survival and quality of life [14-16]. In terms of patient management, pharmacological therapy for T2DM (like metformin) has been proposed as a good strategy to manage CAS, reinforcing the need to study both pathologies simultaneously [17].

Nevertheless, there is currently insufficient information available regarding the molecular mechanisms involved in the development of CAS and its relationship with T2DM. The use of ‘omics techniques like proteomics, metabolomics or transcriptomics, represents a good starting point to better understand these mechanisms, and to select adequate prognostic, diagnostic and therapeutic biomarkers. Recently, we used MS-based proteomics to evaluate the effect of T2DM on calcified and non-calcified AVs from patients with CAS, highlighting the importance of apolipoproteins in this relationship [17]. Such studies are essential to design more appropriate preventive and therapeutic approaches, defining patients at high risk who require regular medical check-ups to monitor the evolution of CAS, and to define the most appropriate time and strategy for treatment.

**Figure 1.**
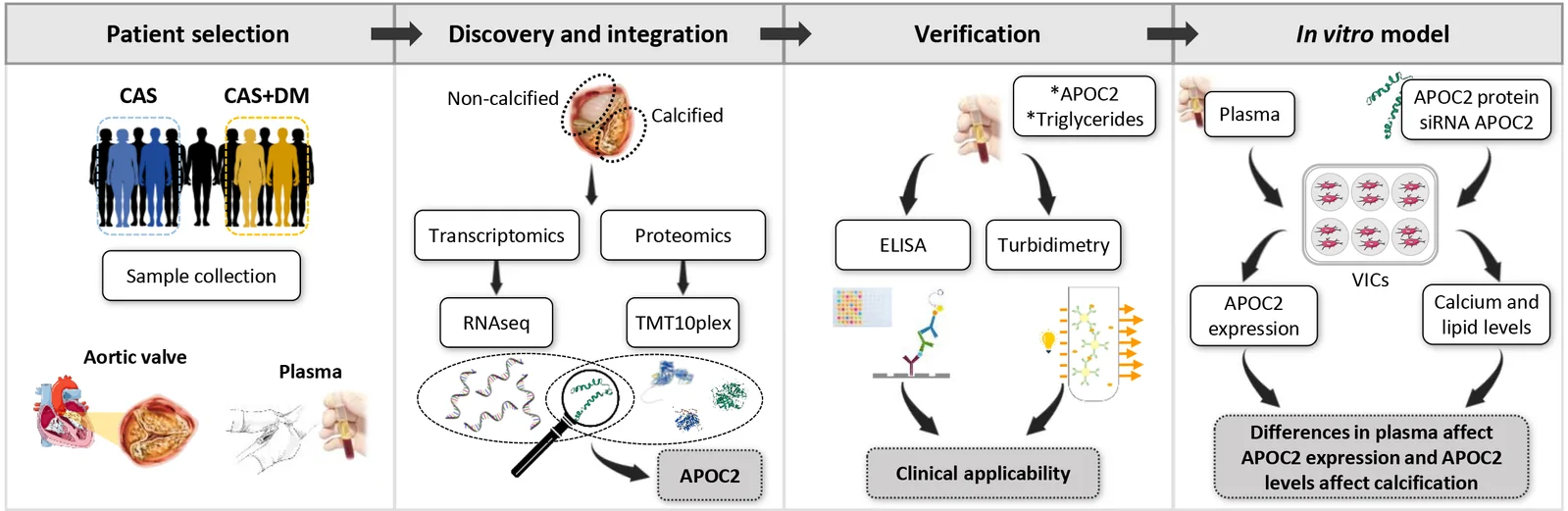
**Experimental pipeline**. Calcified and non-calcified valve tissue from patients with CAS, or with CAS and T2DM, was studied through proteomics and transcriptomics. After the integration of the results, apoC2 was highlighted as a potential biomarker, being measured in plasma samples using ELISA and turbidimetry, and also investigated through the use of an in vitro model to evaluate its therapeutic potential.

Here, we followed an -omics workflow that involved high-throughput RNA sequencing and MS-based proteomics on AV tissue from CAS patients with and without T2DM, which returned the apoC2 protein as a robust biomarker of T2DM in patients with CAS. This protein was then validated in plasma samples from an independent cohort of patients to evaluate its potential diagnostic power. In addition, a cellular model was used to assess the effect of circulating factors in plasma on aortic VICs, as well as the direct effect of apoC2, evaluating its potential as a therapeutic target (Fig. 1). Together, our results underscore the importance of apoC2 in the development of CAS in patients with T2DM, as well as its implication in calcification, suggesting new avenues for research and a potential target for the development of new treatments in the future.

## MATERIALS AND METHODS

### Selection of subjects for the study

Patients with CAS, with and without T2DM, were recruited at the Cardiac Surgery unit of the Hospital Universitario de Toledo (Toledo, Spain) and at the Cardiology Unit of the Hospital 12 de Octubre (Madrid, Spain), and they were classified into 2 groups: a) CAS without T2DM (nDM-CAS); and b) CAS with T2DM (DM-CAS). Aortic valve tissue was obtained from patients who underwent AV replacement and plasma samples were also obtained from them just before surgery. The inclusion criteria established were individuals: over 50 years of age; not currently participating in a clinical trial with drugs at the time of inclusion; a diagnosis of valve involvement or absence thereof, with or without T2DM; and having signed an informed consent (if an individual was unable to provide consent, their legal representative was authorised to provide consent on their behalf). The exclusion criteria for all patients included: refusal to provide signed informed consent; a severe co-morbidity, such as ischemic heart disease with ventricular dysfunction, end-stage chronic kidney disease (stage 5) or on dialysis, familial hypercholesterolemia, secondary arterial hypertension (AHT), cancer, a bicuspid AV, a family or personal history of an aortopathy, moderate or more severe rheumatic or mitral valve disease.

The study was carried out in accordance with the Helsinki Declaration and received a favourable evaluation from the Ethical Committees of the hospitals involved in obtaining samples (CEIC 836 Hospital Universitario de Toledo and CEIm 18/315 Hospital 12 de Octubre). Signed informed consent was obtained from all subjects prior to their inclusion on the study.

### Sample collection

To obtain plasma, blood samples were collected from all the study participants in tubes pretreated with ethylenediaminetetraacetic acid (EDTA) and they were processed no later than 2 hours after collection to avoid degradation. The blood was centrifuged for 10 min at 1125g (5804R, Eppendorf), and the resulting supernatant (plasma) was immediately frozen and stored at -80 °C until use. The AVs from patients with CAS were processed within 2 hours of extraction, first washing them in a saline solution to eliminate any traces of blood and then storing the 3 leaflets at -80 °C until use.

### Experimental overview from the ‘omics to the *in vitro* model

The experimental strategy involved six steps: (i) RNA sequencing, (ii) Tandem Mass Tag (TMT) analysis, (iii) integrative transcriptomic and proteomic analysis, (iv) verification of gene candidates in plasma samples using ELISA and turbidimetry assays, (v) *in vitro* model using plasma from patients as treatment, and (vi) *in vitro* model using recombinant apoC2 and APOC2 siRNA to modulate calcification.

### RNA sequencing

For RNA-Seq transcriptome sequencing, total RNA was isolated from 18 samples obtained from 9 CAS patients, including calcified and non-calcified valve tissue. Four different conditions were established: calcified tissue from patients with CAS and T2DM (DM-Cal); non-calcified tissue from patients with CAS and T2DM (DM-nCal); calcified tissue from patients with CAS without T2DM (nDM-Cal); and non-calcified tissue from patients with CAS without T2DM (nDM-nCal). Total RNA was extracted from the AV tissue using the RNeasy Lipid Tissue Mini kit (Qiagen) and the QIAzol Lysis Reagent following the manufacturer’s instructions. RNA concentrations were measured with a NanoDrop spectrophotometer (NanoDrop ND-1000, Thermo Fisher Scientific) and absorbance ratios (260/280 and 260/230) were also used to assess the purity of the RNA. To evaluate if the quality and quantity of the RNA extracted was suitable for Next Generation Sequencing (NGS), the DV200 values (percentage of RNA fragments >200 nucleotides) were determined using an Agilent Technologies 2100 Bioanalyzer (Agilent Technologies, CA, USA).

RNA libraries were generated with the TruSeq RNA Access Library Prep kit (Illumina, CA, USA), following the manufacturer’s instructions. The quality of the libraries was checked on an Agilent Technologies 2100 Bioanalyzer using a DNA 1000 chip and quantified using Qubit DNA HS Assay kit on a Qubit 2.0 fluorometer (Thermo Fisher Scientific, MA, USA). All the libraries were then sequenced on an Illumina Novaseq6000 platform (Illumina), producing 150 bp paired-end reads. Quality control of the raw sequenced data was assessed with FastQC, and the reads were then trimmed and aligned to the human reference genome (GRCh38). After alignment, the transcripts were quantified using the Salmon algorithm through a standard RNA-Seq pipeline as transcripts per million (TPM).

### Tandem Mass Tag (TMT) analysis

For proteomics, 16 AV samples of Cal and nCal tissue from 8 diabetic and non-diabetic patients were studied, defining the same study groups as in the transcriptomic assay: DM-Cal, DM-nCal, nDM-Cal and nDM-nCal. Details on the proteomic methods and analysis employed have been described previously in detail [17]. Briefly, tissue samples were lysed in 400 µL of lysis buffer (4.44 % SDS, 0.1M Tris) and using stainless steel beads in a bullet blender, and the protein obtained was quantified using a microBCA procedure. The protein from each sample (80 µg) was then digested according to the FASP digestion protocol [18] and labeled for quantification using isobaric tags (TMT: Fisher Scientific) according to the manufacturer’s protocol. The labelled samples were fractionated by high-pH reverse phase chromatography in a HPLC 1100 UV-Vis apparatus (Agilent), with a XBridge Peptide BEH C18 column (130 Å, 5 µm, 2.1 mm x 100 mm: Waters). After fragmentation, labelled peptides were analyzed by nanoLC-ESI-MS/MS using an Orbitrap Fusion Lumos™ Tribrid mass spectrometer (Thermo Scientific), equipped with a Dionex Ultimate 3000 ultrahigh pressure chromatography system (Thermo Scientific) and a TriVersa NanoMate (Advion Inc. Biosciences) nanospray interface. All MS/MS spectra were searched against the SwissProt Human (released 2020_04) database using the Proteome Discoverer v2.3 software and the Sequest-HT search engine. To ensure the accuracy of the MS data analysis, false discovery rates (FDRs) were set to 1%.

### Enzyme-Linked Immunosorbent Assay (ELISA)

The concentration of apoC2 in plasma samples (n=15 samples/group) was determined using a commercial ELISA kit (Elabscience, Ref. H0466), according to the manufacturer’s instructions. The concentration of each protein in the samples was calculated by comparing the optical density values of the samples with the standard curve, performed in duplicate.

### Turbidimetry

Turbidimetry tests were carried out in collaboration with the Clinical Analysis Laboratory at the Hospital 12 de Octubre (Madrid, Spain) using reagents from Randox Laboratories on an automated Biosystem A25 instrument. The technique is based on the formation of an insoluble complex between plasma APOC2 and a specific antiserum, which generates turbidity in the sample. This turbidity is measured at 340 nm, allowing plasma apoC2 levels to be quantified accurately and rapidly.

### Triglyceride assay

Triglyceride assay was performed using the Roche Diagnostic Cobas c701 platform, using an enzymatic colorimetric method. This procedure involves the use of lipoprotein lipase derived from microorganisms to completely and efficiently break down triglycerides into glycerol. Subsequently, the glycerol is oxidised to dihydroxyacetonaphosphate and hydrogen peroxide. In the presence of peroxidase as a catalyst, hydrogen peroxide interacts with 4-aminophenazone and 4-chlorophenol, generating a red dye by an end-point reaction according to Trinder's method. The red colour produced is proportional to the amount of triglycerides present in the sample and is measured photometrically.

### *In vitro* model

Human aortic valvular interstitial cells (HAVICs: Innoprot, P10462) were used here, cells that were isolated from heart valves, cryopreserved in primary cultures and guaranteed to further expand for 10 population doublings under the conditions indicated in the data sheet. As described previously [19], HAVICs were cultured in Fibroblast Medium-2 (FM-2: Innoprot) that is designed for optimal growth of normal human cardiac fibroblasts *in vitro*, and that contained essential and non-essential amino acids, vitamins, organic and inorganic compounds, hormones, growth factors, trace minerals, and a low concentration of fetal bovine serum (FBS, 5%). HAVICs at passage 5 were used in the experiments.

### Cell treatment with patient plasmas

Once the cells had reached the fourth passage they were dissociated with trypsin-EDTA and cultured in 10 cm^2^ 6-well plates (Nunc) for 5 days to analyse the expression of the APOC2. The cells were cultured in a special medium for fibroblasts that favours a quiescent phenotype, named FIB medium which consist of Dulbecco’s Modified Eagle Medium (DMEM: Hyclone) with 2% heat-inactivated FBS, 150 U/mL penicillin-streptomycin, 2 mM L-glutamine, 10 ng/mL fibroblast growth factor (FGF-2), and 50 ng/mL insulin. The medium was supplemented with 5% plasma from the CAS patients, with or without T2DM. After a five-day exposure to the plasma, the cell culture medium was collected, and the cells were lysed. Protein extracts were analysed and APOC2 immunodetection was performed.

### Protein extracts from cell cultures

HAVICs were trypsinized and homogenized in a lysis buffer (LB1) composed of 0.1% SDS, 2.5 mM dithiothreitol (DTT) and 1 mM phenylmethylsulfonyl fluoride (PMSF) in phosphate buffered saline (PBS). After 3 sonication/ice cycles, the lysate was centrifuged and the supernatant (E1) collected. A second lysis buffer (LB2), composed of 7M urea, 2M thiourea and 4% 3-[(3-Colamidopropyl) dimethylammonio]-1-propanesulfonate (CHAPS)), was then added to the pellet recovered and the lysate was again subjected to 3 cycles of sonication/ice. Finally, this lysate was centrifuged and the supernatant obtained (E2) was combined with supernatant E1. The protein concentration in the cell lysates was determined by the Bradford-Lowry method (Bio-Rad) and the lysates were stored at -80 °C until use. Culture medium was used directly without any additional processing other than protein quantification by the Bradford-Lowry method.

### Immunodetection

The proteins obtained from the cell extracts and the culture medium were resolved by SDS-PAGE in a Miniprotean II vertical electrophoresis system (Bio-Rad) and then transferred to a nitrocellulose membrane (Trans-Blot SD Cell BioRad). Adequate protein transfer was confirmed by Ponceau Red S (Sigma) staining, ensuring all the samples had been loaded equally. The membranes were blocked for 1 h in a solution of PBS/0.1% (v/v) Tween20 containing 7.5% (w/v) non-fat dry milk and they were probed overnight with the corresponding primary antibodies against apoC2 (1:100, ab76452: Abcam) and GAPDH (1:1000, #2118: Cell signalling), both diluted in wash buffer containing 5% (w/v) non-fat dry milk. A secondary antibody only control was performed to ensure the specificity of the primary antibodies. After washing, the membranes were incubated for 1 h with the corresponding secondary antibody (1:1000, #7074P2: Cell Signaling), diluted in wash buffer containing 5% (w/v) non-fat dry milk. Finally, the membranes were incubated with the chemiluminescent substrate ECL (GE Healthcare) and the light emitted was detected in the Amersham Imager 600 luminescent image analyser (GE Healthcare). Images were analysed with ImageQuant TL software (GE Healthcare) to quantify the protein bands.

### Recombinant APOC2 treatment of interstitial cells

To analyse the effect of apoC2 in calcification and lipid deposition, HAVICs were first exposed for 5 days to the recombinant apoC2 protein (ELB-PKSH032084, ElabScience) at 2 different concentrations (10 and 20 ng/ml), diluted in 2 different cell culture media: FIB media, to maintain the quiescent phenotype; and osteogenic media (OM: FIB medium supplemented with 5% ascorbate-2-phosphate and 100 nM dexamethasone) to induce osteogenic differentiation.

### APOC2 silencing in interstitial cells

HAVICs were transfected with siRNAs (60 nM) against APOC2 or control siRNAs (ON-TARGETplus SMARTpool siRNA, L-010972-00-0005, and ON-TARGETplus non-targeting pool, 77D-001810-10-05: Cultek) using the LipofectamineTM RNAiMAX transfection reagent (Invitrogen) for siRNA delivery and following the manufacturer’s instructions. After transfection (5h), the medium was replaced, the cells were washed twice in culture medium, and they were then exposed to the corresponding treatment. Finally, the cells were analyzed for calcification and by Quantitative Real-Time PCR.

### Quantitative Real-Time PCR (qRT-PCR)

After recovery with trypsin-EDTA, RNA extraction of HAVICs was performed with the RNeasy Mini Kit (50974104, QIAGEN) following the manufacturer’s indications. Dnase I-treated total RNA (1 µg) was reverse transcribed into cDNA using the High Capacity cDNA Archive Kit (Applied Biosystems, Foster City, CA, USA) and random hexamers. Primers were prepared at a 100 nM concentration (Table 1). To quantify gene expression, RT-PCR was performed using the qPCRBIO SyGreen Mix Hi-ROX SyGreen realtime PCR (K7PB20.12-05, PCR BIOSYSTEMS) on a 7900HT Fast Real-Time PCR (Applied Biosystems), and using glyceraldehyde 3-phosphate dehydrogenase (GAPDH: Invitrogen) as an endogenous control. Each sample was amplified in duplicate and the data were normalized to GAPDH, using 2-ΔCt to calculate the expression of each gene.

**Table 1:** PCR primers used.

Gene	Forward Primer	Reverse Primer
APOC2 - a	5′GTC AGC AAA GAC AGC CGC CCA 3′	5′AGC CCC TCC ATC TTG GCC CTT 3′
APOC2 - b	5′ GTT GGA GAC GAG GCT TAC CA 3	5′ AGC CTC TGG AAT AGC TGG GA 3′
GAPDH	5′GTC TCC TCT GAC TTC AAV AGC G 3′	5′ACC ACC CTG TTG CTG TAG CCA A 3′

### Viability assay

The LIVE/DEADTM assay kit (Cat. No. L3224: Molecular Probes, Life Technologies Corporation) was used to visualize the distribution of viable and non-viable HAVICs after APOC2 silencing or recombinant apoC2 treatment. Briefly, trypsinized cells were washed and resuspended in PBS, and then 2 µL of calcein (50 µM) and 2 µL ethidium homodimer-1 (EthD-1, 2 mM) was added per ml of cell suspension. After a 15 min incubation at room temperature the stained cells were analyzed by flow cytometry on a CytoFLEX S cytometer (Beckman Coulter), using 488 nm excitation, and measuring green fluorescence emission for calcein (i.e., 530/30 bandpass) and red fluorescence emission for ethidium homodimer-1 (i.e., 610/20 bandpass). The flow cytometry data were analyzed with FlowJo 10.8.1 software (BD Biosciences).

### Alizarin Red Staining

The culture medium was removed from the wells, the cells were washed twice with PBS, and they were then fixed for 15 min with paraformaldehyde (PFA, 4%). The cells were then incubated for 10 min with alizarin red (Sigma Aldrich) and then washed with distilled water to remove any excess dye. Calcium deposits were visualized under an Olympus IX83 inverted microscope, and 49 images per well were captured and analysed using Scan^R software. Each experiment was performed in triplicate.

### Oil Red Staining

After removing the culture medium, the cells were washed twice with PBS, fixed for 15 min with 4% PFA and incubated in 60% isopropanol for 5 min. The cells were then stained with Oil Red ‘O’ solution for 30 min and washed with distilled water to remove any excess dye. Lipids were visualized under an Olympus IX83 inverted microscope, and 49 images per well were captured and analysed using Scan^R software. Each experiment was performed in triplicate.

### Statistical analysis

Statistical analyses were performed using JASP v.0.18.1, an open-source statistical analysis software. Normality was assessed with the Shapiro-Wilk test and consequently, normally distributed variables were analyzed using parametric tests and those distributed non-normally were analyzed with non-parametric tests. For comparisons of two groups, Student’s t-test or the Mann-Whitney U test were used for continuous variables and Pearson’s chi-square test for discrete variables. For multiple comparisons and continuous variables, one-way ANOVA and a Tukey’s post hoc analysis was used for normally distributed variables while Kruskal-Wallis test and Dunn’s post hoc analysis was used for non-normal data. Analysis of small sample size (N<6) was performed using non-parametric tests. The descriptive data are presented as the means ± standard deviation (SD) or as percentages. Statististical significance was accepted when *p*-value<0.05 and ROC curves were generated using SPSS 15.0 for windows software (SPSS Inc.).

### Role of the funding source

The funders of this study had no role in the design, data collection, data analysis, data interpretation, or writing of this manuscript.

## RESULTS

### Clinical characteristic of the study population

The study includes 50 patients with CAS: 25 with T2DM and 25 without T2DM. Considering all the patients included in the study, the most prevalent risk factor is hypertension (86%), followed by dyslipidemia (66%) and coronary artery disease (56%). Accordingly, antihypertensive and lipid-lowering drugs are also the most common therapies. As it is shown in Table 2, these patients were divided into 2 cohorts, for the discovery and for the verification phase. The clinical characteristics of the study groups within each cohort were matched in order to avoid differences in main cardiovascular risk factors, with *p*-value>0.05 in all cases, except for T2DM and hypoglycemic drugs.

**Table 2:** **Clinical characteristics of the participants**. Age is expressed as the means ± standard deviation (SD) while discrete variables are presented as percentages.

	Discovery phase			Verification phase		
	nDM-CAS (n=8)	DM-CAS (n=9)	*p*	nDM-CAS (n=17)	DM-CAS (n=16)	*p*
Age, y	74.62±5.37	75.00±3.46	0.865	75.00±6.49	73.12±7.33	0.442
Sex, M/F	37.5/62.5	33.3/66.7	0.858	58.8/41.2	50.0/50.0	0.611
AHT, %	87.5	100.0	0.274	70.6	93.8	0.085
Smoking, %	12.5	33.3	0.312	23.5	18.8	0.737
Dyslipidemia, %	62.5	88.9	0.200	58.8	62.5	0.829
Obesity, %	25.0	44.4	0.402	23.5	18.8	0.737
CAD, %	50.0	77.8	0.323	47.1	56.3	0.598
AHT treatment, %	75.0	100.0	0.110	82.4	93.8	0.316
DL treatment, %	62.5	66.7	0.858	52.9	68.8	0.353
DM treatment, %	0.0	77.8	0.001	0.0	75.0	0.000

Abbreviations: AHT, arterial hypertension; CAD, coronary artery disease; CAS, calcific aortic stenosis; DL, dyslipidemia; DM, diabetes mellitus; DM-CAS, diabetic subjects; M/F, male/female; nDM-CAS, non-diabetic subjects; *p*, *p*-value.

**Figure 2.**
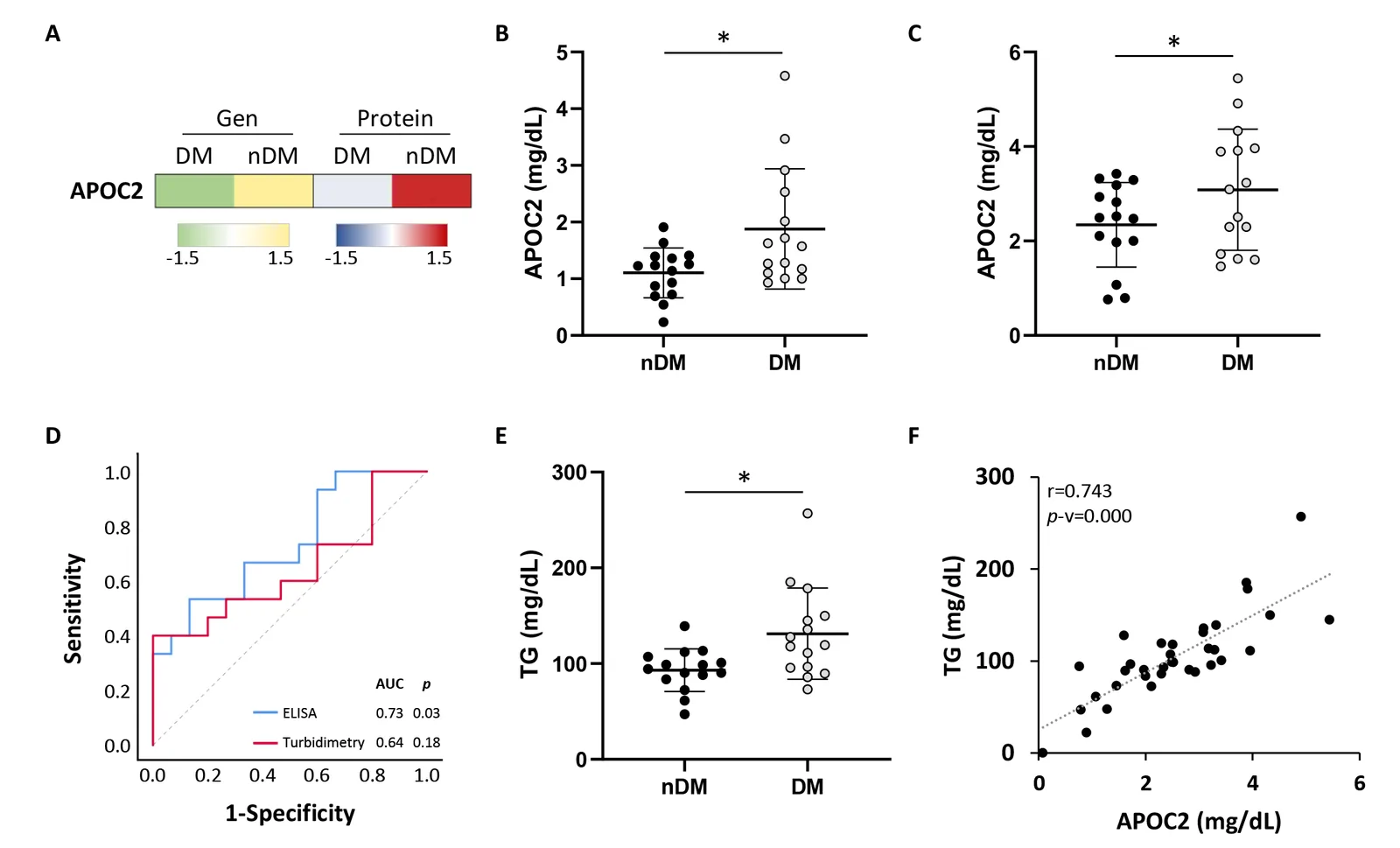
**Tissue and plasma apoC2**. (**A**) Proteomic analysis of the apoC2 protein (n=4 samples/group) and transcriptomic APOC2 gene expression (n=5 samples with T2DM and 4 samples without T2DM) in AV tissue from CAS patients with or without T2DM, showing the calcified:non-calcified ratio. (**B**) ELISA verification of the apoC2 in plasma from patients with CAS, with or without DM (n=15 samples/group, assessing significance with Mann-Whitney U test). (**C**) Turbidimetry verification of apoC2 in plasma samples from patients with CAS, with or without DM (n=15 samples/group, assessing significance with Student’s *t*-test). (**D**) ROC curves of the apoC2 levels assessed by each technique to evaluate the sensitivity and specificity of this potential biomarker. (**E**) Colorimetric measurement of triglycerides (TGs) in plasma from patients with CAS, with or without DM (n=15 samples/group, Student’s *t*-test). F) Correlation between the apoC2 and TG levels. Abbreviations: DM, diabetic patients; nDM, non-diabetic patients; TG, triglyceride. *p-value <0.05.

### APOC2 quantification and differential expression

For the quantitative analysis, the ratio of the values in calcified:non-calcified tissue from each patient was compared. After a statistical analysis of the proteome and transcriptome of the AVs from patients with CAS, with or without T2DM, the results were compared to identify robust biomarkers that may possibly serve for clinical use. These results are publicly available under the BioStudies Accession number S-BSST2097 and can be accessed at https://www.ebi.ac.uk/biostudies/studies/S-BSST2097. We found that both apoC2 protein and the *APOC2 gene* returned a *p*-value <0.05 (*p*-value=0.006 and 0.039, respectively). Also, in both cases the differences between tissue from diabetic and non-diabetic patients was higher than 50%, with a fold change (FC)=-1.72 and -5.07 in APOC2 protein and gene, respectively (Fig. 2A). APOC2 protein and *APOC2 gene* display the same trend in expression (Fig. 2A), suggesting that APOC2 might be a robust biomarker.

### Verification of apoC2 in plasma samples

Turbidimetry and ELISA were used to measure the apoC2 in plasma, revealing higher levels of this protein in diabetic patients with CAS compared to the non-diabetic patients, both through turbidimetry (nDM=2.34±0.89 mg/dL, DM=3.08±1.28 mg/dL; Student’s *t*-test *p*-value=0.038) and ELISA (nDM=1.10±0.44 mg/dL, DM=1.88±1.06 mg/dL; Mann-Whitney U test *p*-value=0.014) (Fig. 2B and C). To evaluate the diagnostic capacity of this marker in the clinic, ROC curves were generated that showed ELISA to be more suitable to measure apoC2 in plasma than turbidimetry, providing a higher AUC (ELISA=0.733, turbidimetry =0.644) and *p*-value (ELISA=0.029, turbidimetry=0.178: Fig. 2D). Higher levels of TGs were also found in diabetic patients with CAS compared to non-diabetic patients (nDM=93.17±22.19 mg/dL, DM=131.20±47.68 mg/dL; Student’s *t*-test *p*-value=0.011: Fig. 2E). As expected, the levels of apoC2 and TGs were positively correlated (r=0.743, *p*-value=0.000: Fig. 2F).

**Figure 3.**
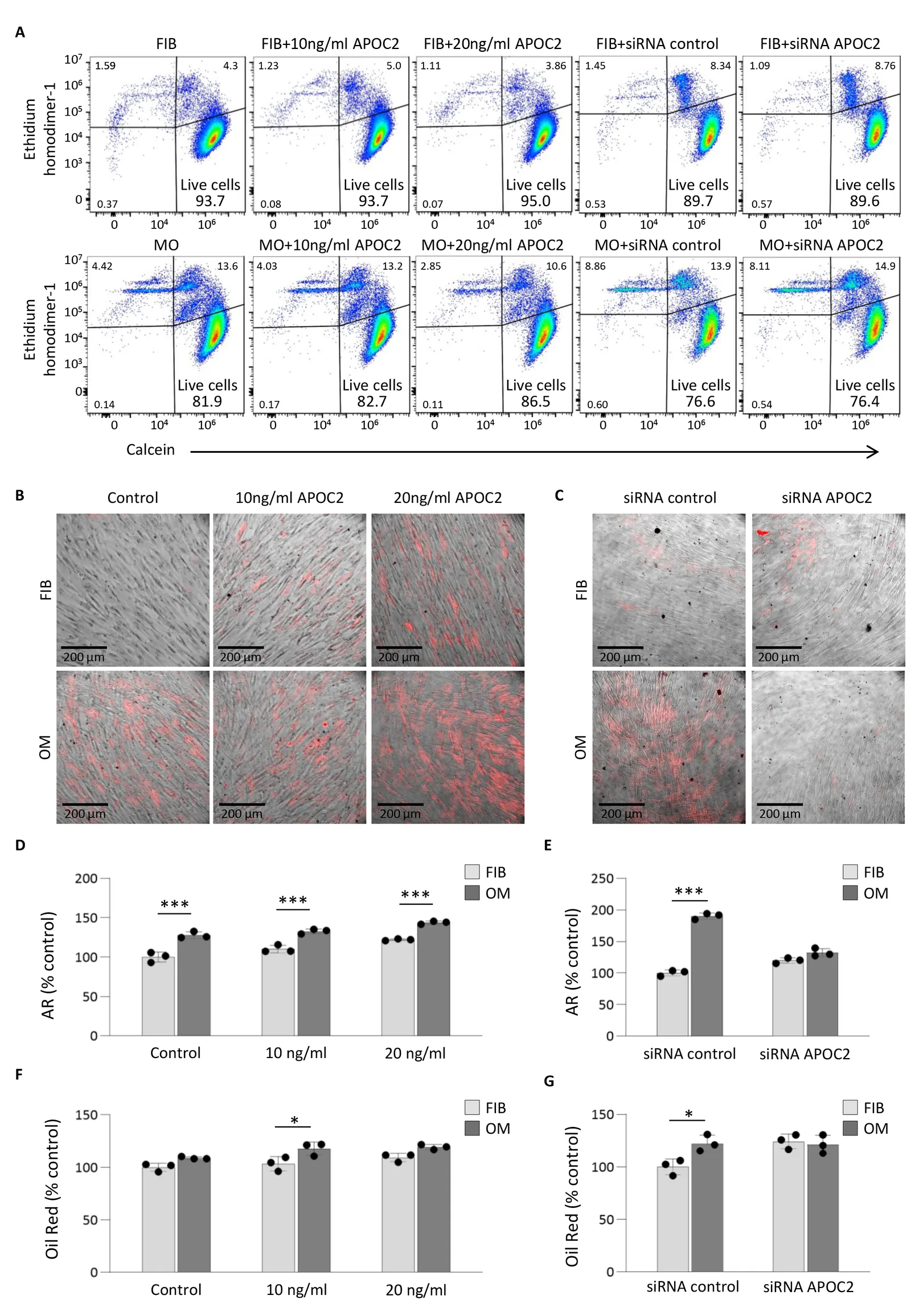
**Effect of apoC2 in HAVICs**. (**A**) Calcein staining and flow cytometry to assess viability, showing the percentage of live cells. (**B**) Alizarin red staining of cells treated with recombinant APOC2. The scale bar corresponds to 200 µm. (**C**) Alizarin red staining of cells treated with APOC2 siRNA. The scale bar corresponds to 200 µm. (**D**) The calcification revealed by alizarin red staining of cells increases as cell are exposed to higher concentrations of recombinant apoC2 (n=3 biological replicates, assessing significance with Kruskal-Wallis and Dunn′s post hoc test). (**E**) The calcification revealed by alizarin red staining is lower in cells in which APOC2 expression is silenced with specific siRNAs, reverting the effect of the OM (n=3 biological replicates, assessing significance with Kruskal-Wallis and Dunn′s post hoc test). (**F**) Oil red staining of cells treated with recombinant APOC2 showing more lipid deposition as the apoC2 concentrations increase (n=3 biological replicates, assessing significance with Kruskal-Wallis and Dunn′s post hoc test). (**G**) Oil red staining shows cells in which APOC2 expression is silenced with specific siRNAs accumulate less lipids, reverting the effect of the OM (n=3 biological replicates, assessing significance with Kruskal-Wallis and Dunn′s post hoc test). Cells cultured in FIB medium without any treatment (neither recombinant apoC2 nor siRNA APOC2) were used as the reference (100%) in Alizarin red and oil red staining. FIB, medium for fibroblasts; OM, osteogenic media. *p-value <0.05, ***p-value<0.001.

### Effect of diabetes and APOC2 expression in HAVICs

To elucidate whether apoC2 has any direct effect on HAVIC calcification and lipid deposition, cells were treated with recombinant apoC2 or siRNA APOC2. The viability of the cells was first established using calcein staining and flow cytometry, with more than 80% of cells remaining alive in all cases (Fig. 3A). As the treatments did not affect cell viability, calcification was examined, and it was seen to increase when cells were treated with recombinant apoC2 (10 or 20 ng/ml: Fig. 3B and D). Cells cultured in FIB medium without any treatment (neither recombinant apoC2 nor siRNA APOC2) were used as the reference (100%). Results showed that calcification significantly increased in all cases, especially in the presence of 20 ng/ml of the recombinant protein (Table 3). Indeed, cells culture in FIB+20 ng/ml of apoC2 underwent a similar increase in calcification (27.66%) as cells cultured in OM (22.01%; *p*-value=0.444). By contrast, cells cultured in OM+APOC2 siRNA underwent significantly less calcification (an increase of 32.05%) than cells cultured in OM alone (an increase of 90.14%) relative to FIB medium as a reference (*p*-value=0.032: Fig. 3C and E).

**Table 3:** Calcification of HAVICs in different culture conditions showing the *p*-values, calculated using Kruskal-Wallis and Dunn’s test.

Condition A	Mean±SD (%)	Condition B	Mean difference (B-A)	*p*-value
FIB	100.00±6.44	FIB+10ng/ml APOC2	10.07	0.491
		FIB+20ng/ml APOC2	22.01	0.169
		OM	27.66	0.032*
		OM+10ng/ml APOC2	32.67	0.007*
		OM+20ng/ml APOC2	43.66	0.001*

FIB+10ng/ml APOC2	110.07±4.90	FIB	-10.07	0.491
		FIB+20ng/ml APOC2	11.94	0.169
		OM	17.59	0.146
		OM+10ng/ml APOC2	22.6	0.047*
		OM+20ng/ml APOC2	33.59	0.006*

FIB+20ng/ml APOC2	122.01±1.16	FIB	-22.01	0.169
		FIB+10ng/ml APOC2	-11.94	0.491
		OM	5.65	0.444
		OM+10ng/ml APOC2	10.66	0.194
		OM+20ng/ml APOC2	21.65	0.039*

OM	127.66±4.40	FIB	-27.66	0.032
		FIB+10ng/ml APOC2	-17.59	0.146
		FIB+20ng/ml APOC2	-5.65	0.444
		OM+10ng/ml APOC2	5.01	0.592
		OM+20ng/ml APOC2	16.00	0.194

OM+10ng/ml APOC2	132.67±3.07	FIB	-32.67	0.007*
		FIB+10ng/ml APOC2	-22.6	0.047*
		FIB+20ng/ml APOC2	-10.66	0.194
		OM	-5.01	0.592
		OM+20ng/ml APOC2	10.99	0.444

OM+20ng/ml APOC2	143.66±1.95	FIB	-43.66	0.001*
		FIB+10ng/ml APOC2	-33.59	0.006*
		FIB+20ng/ml APOC2	-21.65	0.039*
		OM	-16.00	0.194
		OM+10ng/ml APOC2	-10.99	0.444

FIB+siRNA control	100.00±4.67	FIB+siRNA APOC2	19.69	0.308
		OM+siRNA control	90.14	0.002*
		OM+siRNA APOC2	32.05	0.042*

FIB+siRNA APOC2	119.69±4.29	FIB+siRNA controls	-19.69	0.308
		OM+siRNA control	70.45	0.042*
		OM+siRNA APOC2	12.36	0.308

OM+siRNA control	190.14±4.76	FIB+siRNA controls	-90.14	0.002*
		FIB+siRNA APOC2	-70.45	0.042*
		OM+siRNA APOC2	-58.09	0.308

OM+siRNA APOC2	132.05±6.41	FIB+siRNA controls	-32.05	0.042*
		FIB+siRNA APOC2	-12.36	0.308
		OM+siRNA control	58.09	0.308

Lipid deposition was also examined through oil red staining (Table 4), with greater deposition evident in cells cultured in the presence of recombinant apoC2 (Fig. 3F) relative to those maintained in FIB medium as a reference. The increase in lipid deposition was especially favoured in the presence of OM and in the presence of apoC2 (20 ng/ml), with an increase of 19.32% (*p*-value=0.004). Importantly, there were also differences in the cells cultured in OM (Fig. 3G) relative to those maintained in FIB, with an increase of 15% (*p*-value=0.013) that was reverted when APOC2 expression was silenced with the specific siRNAs (*p*-value=0.651).

**Figure 4.**
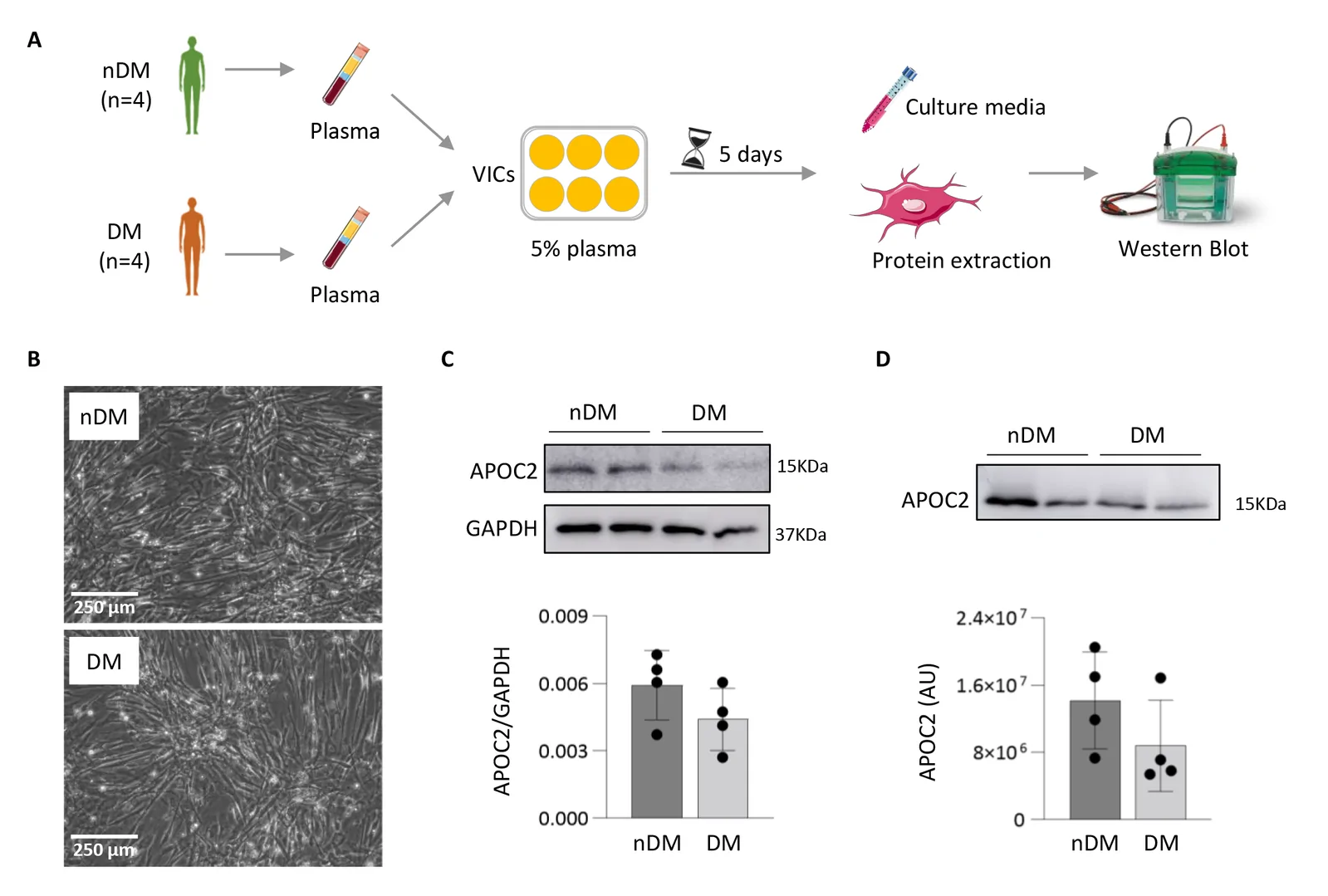
**Effect of plasma from diabetic and non-diabetic patients with CAS in HAVICs**. (**A**) Schematic workflow. (**B**) Microscope images of VICs treated with plasma from diabetic and non-diabetic patients with CAS. (**C**) Immunodetection of APOC2 in protein extracts from VICs (n=4 biological replicates/group, assessing significance with Mann-Whitney U test). APOC2 expression is normalized to GAPDH levels. (**D**) Immunodetection of APOC2 in culture media (n=4 biological replicates/group, assessing significance with Mann-Whitney U test).

To assess if circulating factors might affect the apoC2 in HAVICs, cell cultures were exposed for 5 days to plasma from patients with CAS, with and without diabetes (Fig. 4A and B). There was less intracellular APOC2 in cells exposed to plasma from diabetic patients than that produced in the presence of plasma from non-diabetic patients (nDM=0.006±0.0015 a.u., DM=0.004±0.0014 a.u.; *p*-value=0.343 normalized to GAPDH levels), as also occurred in the calcified AV tissue (Fig. 4C). By contrast, there was less apoC2 in the cell culture medium after the 5-day exposure to plasma from patients with CAS and T2DM (nDM=14190472.73±5771703.97, DM= 8809084.81±6133204.10; *p*-value=0.114: Fig. 4D), the opposite effect to that seen when APOC2 was measured directly in the plasma samples, suggesting a strong response of HAVICs.

**Table 4:** Results of lipid deposition in HAVICs under different culture conditions showing the *p*-values calculated using Kruskal-Wallis and Dunn’s test.

Condition A	Mean±SD (%)	Condition B	Mean difference (B-A)	*p*-value
FIB	100.00±3.82	FIB+10ng/ml APOC2	3.42	0.541
		FIB+20ng/ml APOC2	9.33	0.079
		OM	8.79	0.194
		OM+10ng/ml APOC2	17.83	0.005*
		OM+20ng/ml APOC2	19.32	0.004*

FIB+10ng/ml APOC2	103.42±6.72	FIB	-3.42	0.541
		FIB+20ng/ml APOC2	5.91	0.251
		OM	-5.37	0.491
		OM+10ng/ml APOC2	14.41	0.027*
		OM+20ng/ml APOC2	15.90	0.022*

FIB+20ng/ml APOC2	109.33±3.90	FIB	-9.33	0.079
		FIB+10ng/ml APOC2	-5.91	0.251
		OM	-0.54	0.646
		OM+10ng/ml APOC2	8.50	0.284
		OM+20ng/ml APOC2	9.99	0.251

OM	108.79±1.18	FIB	-8.79	0.194
		FIB+10ng/ml APOC2	5.37	0.491
		FIB+20ng/ml APOC2	0.54	0.646
		OM+10ng/ml APOC2	9.04	0.126
		OM+20ng/ml APOC2	10.53	0.108

OM+10ng/ml APOC2	117.83±6.30	FIB	-17.83	0.005*
		FIB+10ng/ml APOC2	-14.41	0.027*
		FIB+20ng/ml APOC2	-8.50	0.284
		OM	-9.04	0.126
		OM+20ng/ml APOC2	1.49	0.939

OM+20ng/ml APOC2	119.32±2.39	FIB	-19.32	0.004*
		FIB+10ng/ml APOC2	-15.90	0.022*
		FIB+20ng/ml APOC2	-9.99	0.251
		OM	-10.53	0.108
		OM+10ng/ml APOC2	-1.49	0.939

FIB+siRNA control	100.00±6.87	FIB+siRNA APOC2	3.82	0.651
		OM+siRNA control	15.06	0.013*
		OM+siRNA APOC2	5.93	0.365

FIB+siRNA APOC2	103.82±4.23	FIB+siRNA control	-3.82	0.651
		OM+siRNA control	11.24	0.042*
		OM+siRNA APOC2	2.11	0.651

OM+siRNA control	115.06±2.83	FIB+siRNA control	-15.06	0.013*
		FIB+siRNA APOC2	-11.24	0.042*
		OM+siRNA APOC2	-9.13	0.113

OM+siRNA APOC2	105.93±7.22	FIB+siRNA control	-5.93	0.365
		FIB+siRNA APOC2	-2.11	0.651
		OM+siRNA control	9.13	0.113

## DISCUSSION

In this study, we employed an ‘omics workflow that involved high-throughput RNA sequencing and MS-based proteomics to analyse AV tissue from CAS patients with and without T2DM, confirming the apoC2 protein to be a robust biomarker of T2DM in these patients. This protein was then validated in plasma samples from an independent cohort to evaluate its potential diagnostic power. Moreover, in a cell model we assessed the effect of circulating plasma on the levels of intracellular apoC2 in HAVICs, observing less intracellular apoC2 in cells treated with plasma from patients with CAS and T2DM than with that from patients without T2DM. Finally, HAVICs were treated with recombinant apoC2 or APOC2 siRNAs and the extent of calcification and lipid deposition was seen to be affected, concluding that apoC2 may have an important influence on the development of CAS.

Apolipoproteins are amphipathic and multifunctional proteins that regulate the transport and distribution of lipoproteins, promote the binding of lipoproteins to cell surface receptors, enhance the lipid uptake capacity of cells and activate lipid enzymes [20, 21]. In relation to lipid metabolism, apoC2 activates lipoprotein lipase (LPL) by bringing lipid substrates to the active site of LPL and acting as a cofactor, with LPL activation necessary for TG hydrolysis. Nevertheless, an excess of apoC2 in plasma, as found here, impairs LPL activity and is associated with an increase in TGs [22]. Our results also show a significant increase of plasma TGs in diabetic compared to non-diabetic patients, although its diagnostic power should be further investigated. It should be noted that the diagnostic accuracy is moderate when using either ELISA or turbidimetry, although it is higher with ELISA. An independent study using a larger cohort would be useful in confirming the utility of apoC2 as a diagnostic marker, with potential application in clinical practice. Furthermore, apoC2 and TG levels were positively and significantly correlated. Higher plasma levels of apoC2 and TGs have been seen previously in diabetic patients and related to complications due to diabetes and its relationship with cardiovascular disease (CVD), indicating that this protein may play an important role in CV events [23]. Interestingly, and like excess apoC2, a deficiency or loss of function of apoC2 can also provoke hypertriglyceridemia. The increase in apoC2 has also been associated with alterations to reverse cholesterol transport, necessary to avoid cholesterol accumulation in peripheral tissues through its uptake and transport to the liver and steroidogenic tissues [22, 24].

Although apoC2 was discovered 50 years ago, interest in this protein is resurging due to its importance in the metabolism of TGs and its relationship with CVD, especially atherosclerosis [25-27]. Recently, isoforms of APOC2 have been associated with coronary heart disease and the accumulation of coronary artery calcium in the context of a Multi-Ethnic Study of Atherosclerosis [26]. However, there has been little research into the role of apoC2 in CAS. CAS patients have higher levels of circulating apoC2 than patients with AV regurgitation, such that its alteration occurs in response to AV calcification and not due to the effects of tissue dysfunction [28]. It is also known that apolipoproteins including apoC2 contribute to a prothrombotic fibrin clot phenotype in non-diabetic patients with severe CAS, suggesting that they could affect fibrin clot structure [29]. This phenomenon may involve activation of blood coagulation through TG-rich lipoproteins that contain apoC2 [30]. Remarkably, patients with T2DM also have a prothrombotic phenotype of fibrin clot [31], suggesting that this mechanism may be implicated in the rapid development of CAS in patients with T2DM.

To assess whether apoC2 has a direct effect on calcification and lipid deposition, HAVICs were treated with recombinant apoC2 protein and with siRNAs designed to silence APOC2 expression. Increasing the apoC2 concentration in the cell culture media produces stronger calcification, whereas silencing apoC2 reverts the effect of OM. Less lipid deposition is also observed when apoC2 is silenced, although this effect is less pronounced than when it occurs in conjunction with calcification. Hence, apoC2 is potentially an important therapeutic target to reduce the risk of CAS, especially in patients with T2DM. There is considerable interest at present in apolipoprotein-based mimetic peptides, which have been shown to improve lipid profiles and exert anti-atherosclerotic effects [32]. Although there are relatively few studies into apoC2 mimetic peptides, some are currently underway. For example, 18A-CII is a potent activator of LPL that promotes the *ex vivo* lipolysis of TGs in plasma from patients with apoC2 deficiency, and it markedly lowers plasma TG concentrations in a mouse model of apoC2 deficiency [33-36]. More recently, a second generation apoC2 mimetic peptide called D6PV was described that not only activates LPL but also, it lowers apolipoprotein C-III levels and reduces TG levels. Several steps are needed to reach new therapeutic targets with clinical applications, including the preclinical utilization of animal models prior to transitioning to human clinical trials. Anyway, extending the information available about the effects of these kinds of compounds could pave the way to develop new therapeutic options that may not only improve lipid profiles but also, T2DM and other comorbidities like CAS or other CVDs.

Finally, we assessed if the differences in the composition of plasma from diabetic and non-diabetic patients affects APOC2 expression *in vitro*. This protein is known to be secreted into the plasma and consequently, there have been few studies into intracellular apoC2. However, it is known that the mechanisms regulating APOC2 gene expression differ in distinct cell types, such as hepatocytes, macrophages or enterocytes, suggesting different physiological roles for apoC2 in different tissues [22]. Here, we found that cells treated with plasma from diabetic patients with CAS had less apoC2 that those treated with plasma from non-diabetic patients. Surprisingly, we also saw this tendency in the culture media, even when it is supplemented with 5% plasma. This result suggests that APOC2 regulation possibly differ in patients with CAS, depending on the existence of T2DM, which may be due to differences in the lipid profile or in cofactor concentrations. As diabetic patients have higher TG levels, an over-consumption of apoC2 may occur, leading to lower protein levels in the *in vitro* model. It is known that the rate of apoC2 production is associated with TG concentrations [37], such that our results suggest diabetic patients may not respond in the same way as non-diabetic patients. Either excess or deficient apoC2 levels will cause a dysregulation of TG metabolism, which may be related to different CVDs like atherosclerosis [22]. Taking into account our overall results, we hypothesize that apoC2 metabolism may be altered in these patients, firstly due to a dampened response that is compensated for by the overproduction of apoC2. If we were to better understand this dynamic, we could perhaps prevent that excessive accumulation of apoC2 that, according to our *in vitro* model, may play an important role in AV calcification. This would have important implications in delaying the development of CAS in patients with T2DM.

The main limitation of this study is the relatively small sample size. Nevertheless, this sample size is usual in -omics studies, especially when talking about tissue studies, which usually follow several phases in which the number of individuals increases from a few to many, whereas the number of candidates decreases from hundreds or thousands to just a few: the triangular strategy. Importantly, the discovery phase presented here is performed in aortic valve tissue, which is only obtained through surgeries. In addition, although there are not significant differences among groups, the existence of comorbidities could not be avoided, due to their elevated prevalence in patients of advanced age. Further studies including larger cohorts are recommended to confirm these results. In this context, multicenter cohorts facilitate to obtain large and heterogeneous cohorts, usually providing results with high scientific quality. Nevertheless, the combination of the omics approach with the in vitro study highlights the putative role of APOC2 protein in valve calcification, especially in patients with T2DM.

To the best of our knowledge, this study is the first to provide evidence of differences in plasma apoC2 levels in samples from CAS patients with and without T2DM. Moreover, we demonstrate that elevated apoC2 may play a role in valve cells calcification, paving the way to further research focused on this metabolic pathway and to the possibility of developing new therapeutic approaches in the future. Our results also suggest the importance of early diagnosis as it may prevent irreversible damage from occurring. Having complete profiles of each patient is essential to ensure holistic and personalized clinical management, particularly as complex diseases affect different organs and systems. Given the interconnected nature of body systems, addressing hyperglycemia and insulin resistance in diabetic patients is essential, as they are key triggers of subsequent complications. However, understanding the specific pathways affected in each tissue may help to address disease mechanisms beyond glycemic control, offering a more integrated therapeutic approach. For that reason, traditional medicine must move forward towards precision medicine, which advocates holistic and personalized treatments based on clinical signs, as well as on molecular profiles, the environment or the patient's lifestyle.

## Data Availability

All data generated or analysed during this study are included in this published article and its supplementary information files.
